# Supplementary material for: The solution surface of the Li-Stephens haplotype copying model
Source: Algorithms Mol Biol. 2023 Aug 9;18:12. doi: 10.1186/s13015-023-00237-z (PMC10410957; doi:10.1186/s13015-023-00237-z)
Supplement: Supplementary file 1 — Additional file 1. Additional figures. [file 13015_2023_237_MOESM1_ESM.pdf]

Additional Figures

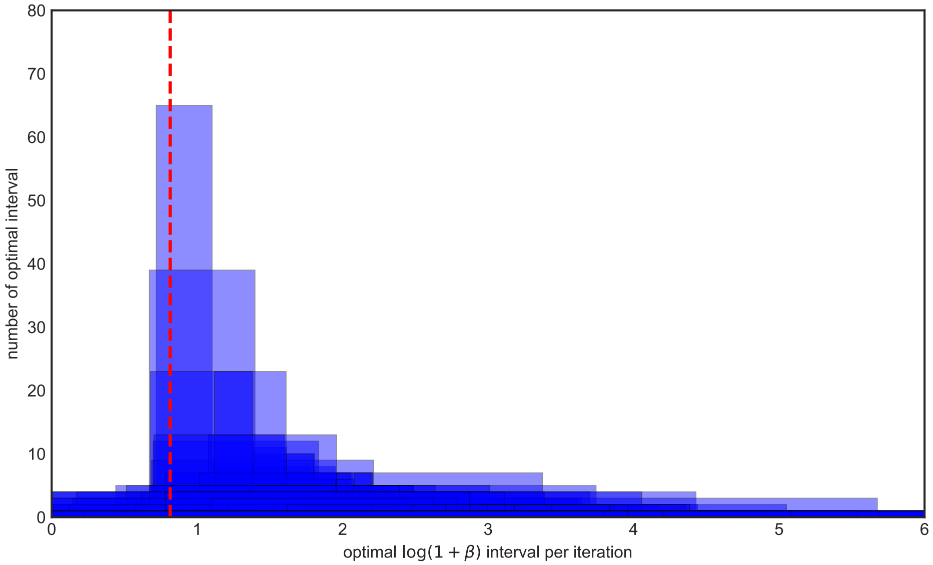

Figure S1 The histogram of optimal β intervals for prephased imputation under the Hamming loss, the x axis is the value of optimal $\log(1+\beta)$ intervals in each iteration, and the y axis is the number of replicates in 1000 iterations. The x axis of the red dash line is the value of $\log(1+\beta_0)$ where β_0 is the truth.

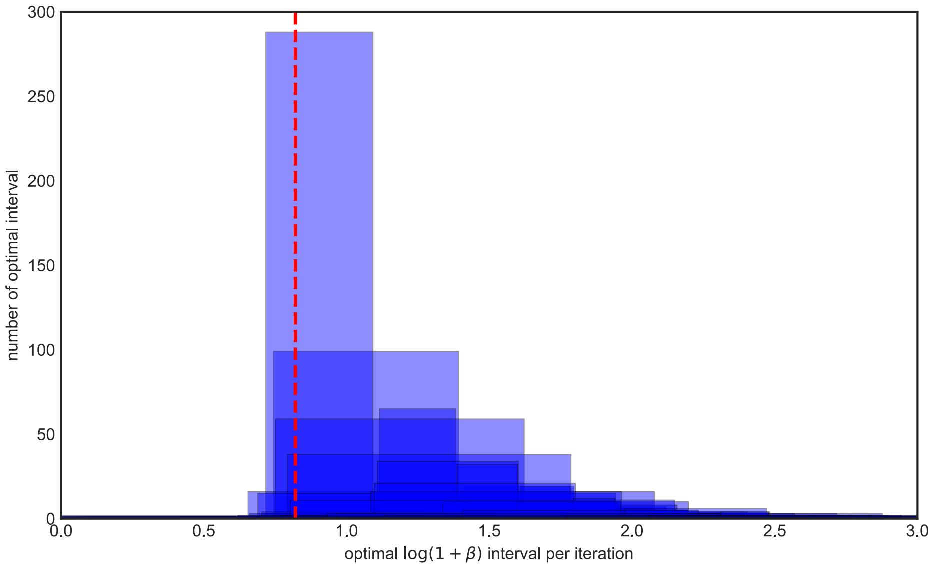

Figure S2 The histogram of optimal β intervals for prephased imputation under the Hamming loss where the model is `Africa_1T12`, the x axis is the value of optimal $\log(1+\beta)$ intervals in each iteration, and the y axis is the number of replicates in 1000 iterations. The x axis of the red dash line is the value of $\log(1+\beta_0)$ where β_0 is the truth.

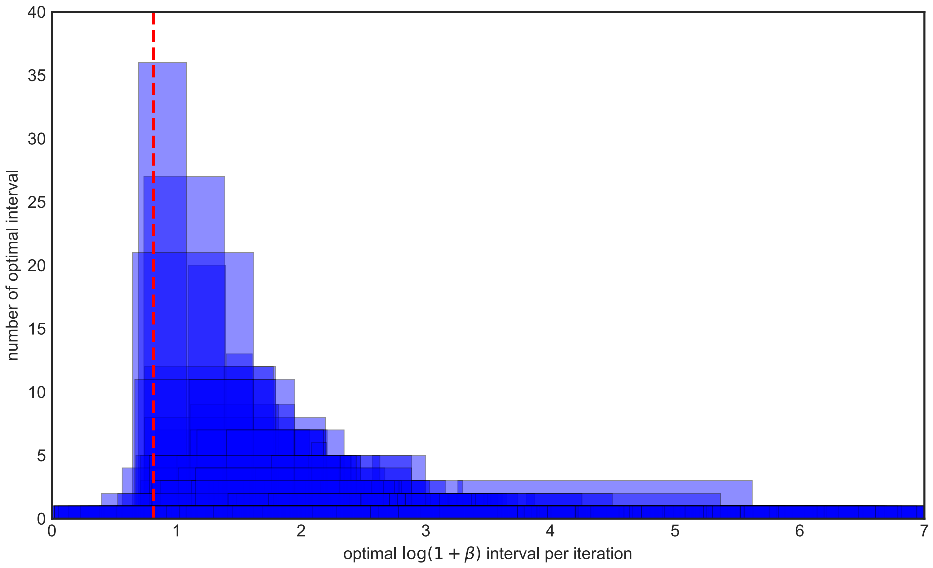

Figure S3 The histogram of optimal β intervals for prephased imputation under the variance-weighted Hamming loss, the x axis is the value of optimal $\log(1+\beta)$ intervals in each iteration, and the y axis is the number of replicates in 1000 iterations. The x axis of the red dash line is the value of $\log(1+\beta_0)$ where β_0 is the truth.

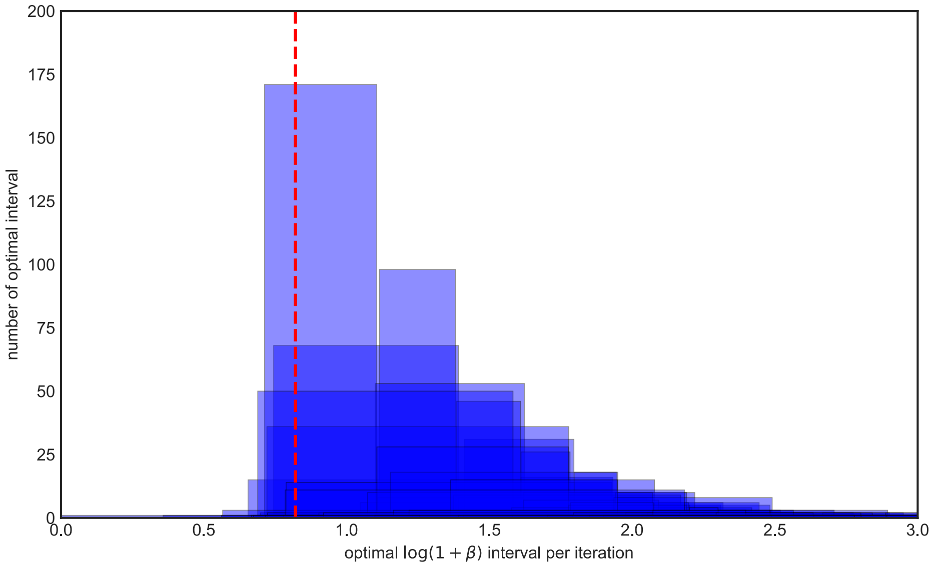

Figure S4 The histogram of optimal β intervals for prephased imputation under the variance-weighted Hamming loss where the model is `Africa_1T12`, the x axis is the value of optimal $\log(1+\beta)$ intervals in each iteration, and the y axis is the number of replicates in 1000 iterations. The x axis of the red dash line is the value of $\log(1+\beta_0)$ where β_0 is the truth.
